# Rituximab-Induced Acute Coronary Syndrome

**DOI:** 10.7759/cureus.94202

**Published:** 2025-10-09

**Authors:** Paschal O Ndukwu, Niharika Patlolla, Neharika Narayanan, Muhammad Mohsin Isar

**Affiliations:** 1 Acute Medicine, United Lincolnshire NHS Hospital Trust, Boston, GBR

**Keywords:** acute coronary syndrome, cardiac chest pain, distal left anterior descending artery (lad), pro-inflammatory cytokines, rituximab therapy

## Abstract

Rituximab is a chimeric monoclonal antibody that selectively targets CD20+ B lymphocytes, making it an effective therapeutic agent in managing diseases where B cells play a critical role in the pathogenesis. The depletion of B lymphocytes is a crucial therapeutic effect in treating both lymphoproliferative disorders and autoimmune diseases.

We present the case of an elderly male with splenic marginal zone lymphoma who developed acute chest pain during rituximab chemotherapy infusion, raising suspicion for rituximab-induced acute coronary syndrome (ACS). Despite an initial rise in cardiac biomarkers (troponin T), the electrocardiography showed no significant changes.

The chest pain resolved promptly after discontinuing the rituximab infusion. Due to a low platelet count, primary percutaneous coronary intervention (PCI) was not performed, and instead, a computed tomography (CT) coronary angiogram was used to confirm the ACS diagnosis. The patient was treated conservatively, and rituximab therapy was temporarily halted.

This case emphasizes the importance of considering the potential cardiotoxic effects of rituximab, particularly in patients with pre-existing cardiovascular risk factors.

## Introduction

Chemotherapy is a vital treatment for many cancers, but it can also lead to significant cardiovascular complications, including myocardial infarction (MI), heart failure, hypertension, and conduction abnormalities [1,2].

Rituximab, a monoclonal antibody that targets CD20, is commonly used to treat various B-cell lymphomas, including splenic marginal zone lymphoma. While rituximab is generally well-tolerated, there have been rare reports linking it to the development of non-ST elevation MI (NSTEMI) [1,3,4].

In this report, we present the case of an elderly male who developed NSTEMI, a type of acute coronary syndrome (ACS), during rituximab infusion for lymphoma treatment, highlighting the need for awareness of this potential cardiotoxic complication.

## Case presentation

An 82-year-old male with a diagnosis of splenic marginal zone lymphoma was undergoing his second cycle of chemotherapy; his initial vital signs were stable prior to the infusion and were documented as follows: blood pressure 144/71 mmHg, pulse 67 beats per minute, respiratory rate 20 breaths per minute, temperature 36.5°C, and oxygen saturation 99% on room air with a National Early Warning Score (NEWS) of 0. However about one-third through the rituximab infusion, he experienced sudden, severe chest pain, rated 8 out of 10 on the pain scale (10 being the maximum pain). The pain was associated with sweating, clamminess, and a sensation of heat, but was localized to the left chest and was non-radiating. It did not change with position, nor was it associated with syncope or palpitations. A repeat of his vital signs immediately showed he now scored 5 on NEWS as his vital signs worsened with significant drop in his pulse rate (Table 1); and this triggered the call for the Medical Emergency Team (MET).

**Table 1:** Vital Signs During Chest Pain CNS, Central Nervous System; O2, Oxygen; NEWS, National Early Warning Score

VITAL SIGNS	
Pulse (beat per minutes)	40
Respiration (breath per minutes)	22
Temperature (Celsius)	36.5
Systolic BP (mmHg)	121
Diastolic BP (mmHg)	74
O2 Saturation (%)	96
On oxygen	no
CNS response	Alert
Pain score	8
Hypercapnia scale 2	no
NEWS score	5

**Figure 1:**
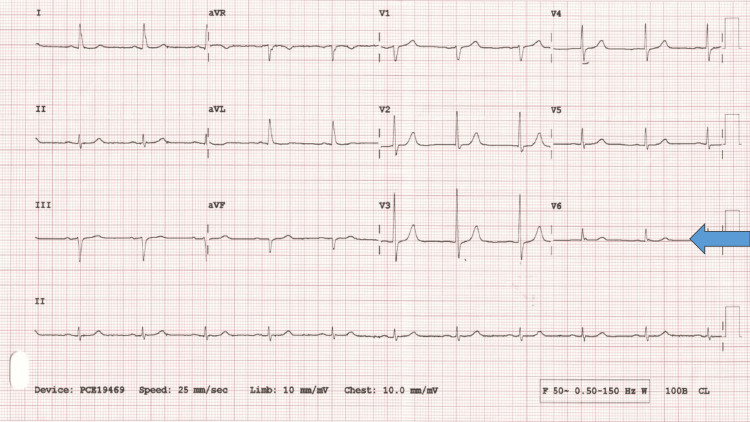
Normal Echocardiogram (ECG) Arrow indicates no ST elevation

As the medical emergency team reviewed him, the infusion was stopped immediately. An urgent electrocardiogram (ECG) was requested (Figure 1), which was normal (no ST elevation), but the routine blood test (Table 2) showed a significant rise in troponin, which worsened four hours later before it began to plummet as shown below.

**Table 2:** Routine Blood Test Showing Rise in Troponin T GFR, Glomerular Filtration Rate; CRP, C-reactive Protein; ALT, Alanine Transaminase; ALP, Alkaline Phosphatase; GGT, Gamma-Glutamyl Transferase

INVESTIGATIONS	REFERENCE RANGE / UNITS		TEST #1	TEST #2: 4 hrs later	TEST #3: 24 hrs later
Sodium	133 - 146 mmol/L		142		
Potassium	3.5 - 5.3 mmol/L		5.2		
Urea	2.5 - 7.8 mmol/L		10		
Creatinine	59 – 104 umol/L		210		
GFR	48 – 72 mL/min		24		
Troponin	0 – 5ng/L		56	199	43
CRP	0 – 5 mg/L		5.8		
ALT	0 – 41 U/L		7		
ALP	30 – 130 U/L		46		
GGT	20 – 40 U/L		26		
Total Protein	60 – 80 g/L		60		
Albumin	35 – 50 g/L		34		
Hemoglobin	132 – 170 g/L		135		
White cells	4.3 – 11.2 10^9/L		12.9		
Platelets	150 – 400 10^9/L		54		
Blood film		Thrombocytopenia & reactive lymphocytes			

**Figure 2:**
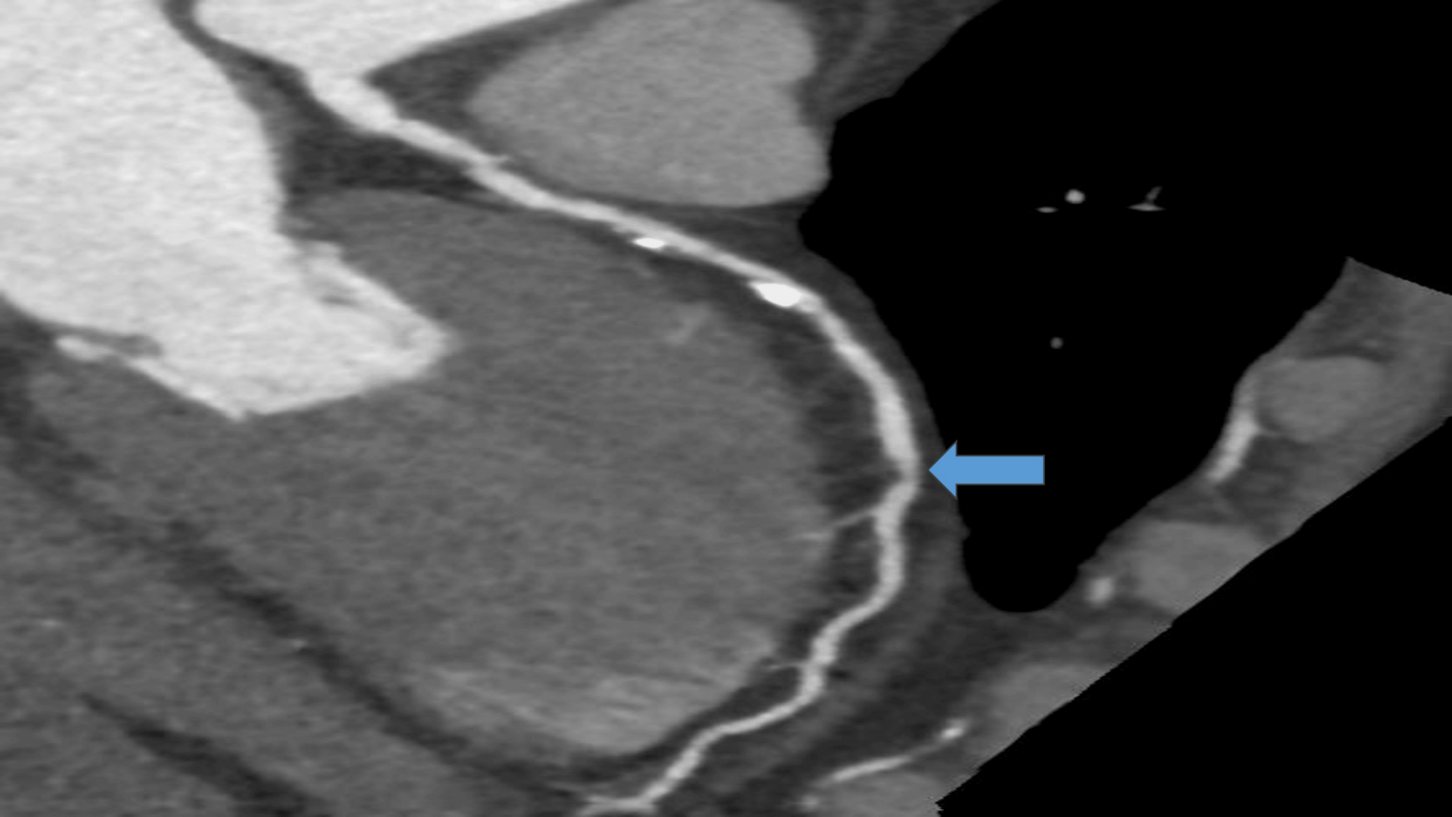
CT-Coronary Angiogram The arrow indicates moderate stenosis in the left anterior descending artery CT, Computed Tomography

Simultaneously, the chest pain began to subside and within 20 minutes after the rituximab infusion was stopped, he no longer reported chest pain. He was transferred to the Emergency Department for further management. On examination, he was conscious, well oriented, Glasgow Coma Scale (GCS) 15/15, no pedal oedema, no neurological deficit, pupils were equal and reactive to light, chest was clear on auscultation, heart sounds were normal, pulse was regular and abdomen was soft with no area of tenderness. An urgent chest X-ray was performed and reported as normal. Due to his low platelet count (54 x 10^9/L) and poor renal function as reported in Table 2 above, a primary percutaneous coronary intervention (PCI) was deferred. Instead, a CT coronary angiogram was performed, which confirmed the diagnosis of ACS with a stenosis to the left descending artery as indicated by the arrow in Figure 2 below.

**Figure 3:**
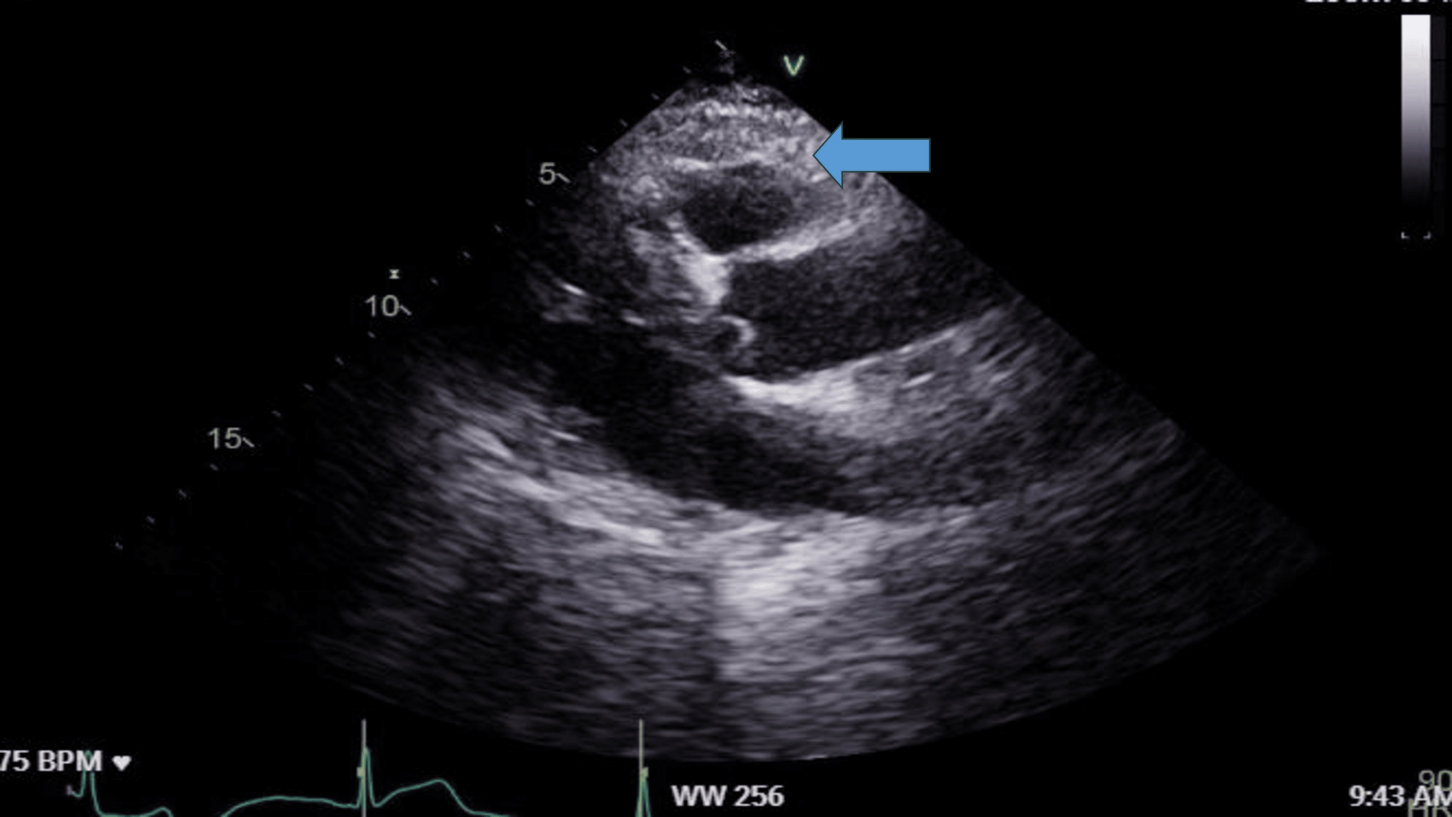
Normal Echocardiogram Arrow indicates normal RV RV, Right ventricle

The findings of the coronary angiography above showed calcium in the proximal and mid-segment of the left anterior descending artery (LAD), mixed plaque in the ostium without significant stenosis, and diffuse atheromatosis in the rest of the vessel with moderate rather than severe stenosis in the middle of the vessel. He subsequently underwent an echocardiogram which excluded any regional wall abnormality in the left ventricle with an ejection fraction of >55% and normal right ventricle wall as shown in Figure 3.

His past medical history included hypertension, chronic kidney disease (CKD), gout, inflammatory arthritis, and a partial nephrectomy for a malignant left kidney tumor. Apart from having been diagnosed with hypertension, he had no other cardiovascular risk factor, except for a family history of sudden death of his brother following a cardiac event. On further assessment, his History, ECG, Age, Risk factors, Troponin (HEART) score fell within an intermediate risk of major adverse cardiac event (MACE) [5,6] as shown in Table 3.

**Table 3:** History, ECG, Age, Risk factors, Troponin (HEART) Score For Major Cardiac Event Predicts six-week risk of major adverse cardiac event in patients with cardiac chest pain 0-3 points => Low risk of MACE 4-6 points => Intermediate risk of MACE 7-10 points => High risk of MACE Risk Factors include: Hypercholesterolemia, Hypertension, Diabetes Mellitus, Cigarette Smoking, Positive Family History, Obesity (BMI >30) or a history of significant atherosclerosis [5,6]. ECG, Electrocardiogram; BMI, Body Mass Index; MACE, major adverse cardiac event

HEART Score		Points	Patient Score
History	Highly Suspicious	2	1
	Moderately Suspicious	1	
	Slightly Suspicious	0	
ECG	Significant ST depression	2	0
	Non-specific ST changes	1	
	Normal	0	
Age	>65 years	2	2
	45-65 years	1	
	<45 years	0	
Risk factor	3 or more risk factors	2	1
	1-2 risk factors	1	
	No risk factor	0	
Troponin	>3 times normal limit	2	2
	1-3 times normal limit	1	
	< normal limit	0	
Total			6

He further mentioned that since starting the second cycle of chemotherapy, he would often experience occasional bouts of chest pain that would quickly resolve in time but this instance is the worst experience he has ever had. His routine medications included febuxostat 80 mg once daily (OD), finasteride 5 mg OD, Fultium-D3 800 units OD and tamsulosin 400 mcg OD. He was noted to have no known drug allergies. 

In terms of his social history, he never smoked cigarettes but consumed alcohol moderately, about six pints per week. He is a retired driller of water and at the time was living with his partner.

## Discussion

Rituximab-induced ACS (NSTEMI), though rare, is a serious complication that should be considered, especially in patients with underlying cardiovascular risk factors [7-9]. 

Rituximab is believed to be therapeutically effective, but it can also lead to a range of cardiovascular complications such as cardiac dysrhythmias, stable angina, unstable angina, cardiogenic shock, hypotension and myocardial infarction [9,10]. These adverse events are primarily driven by cytokine-mediated mechanisms triggered during infusion [8]. Elevated levels of pro-inflammatory cytokines, particularly interleukin-6 (IL-6) and tumor necrosis factor-alpha, can result in systemic symptoms such as fever, rigors, hypotension, and bronchospasm. In some patients, this acute inflammatory response could extensively affect the cardiovascular system, leading to various complications [11,12]. While the exact pathophysiological pathways remain unclear, cytokine-mediated cardiotoxicity coupled with electrophysiological disturbances have been highlighted [10]. 

It is postulated that the rapid cytokine surge during rituximab infusion leads to endothelial dysfunction, vasoconstriction, and even a pro-thrombotic vascular state [8,13]. In patients with underlying atherosclerosis, this inflammatory milieu can destabilize vulnerable plaques, causing rupture or dissection. The exposed plaque surface promotes platelet aggregation and thrombus formation, resulting in coronary occlusion and myocardial infarction. Additionally, cytokine-induced vasoconstriction increases oxygen demand and further exacerbates ischemia [10]. 

In our index patient, the fact that the chest pain resolved shortly after stopping the rituximab infusion and the subsequent rise in cardiac biomarkers strongly suggested that the ACS (NSTEMI) was induced by rituximab. 

The patient received an Aspirin loading dose of 300 mg, followed by 75 mg daily, and a single dose of enoxaparin 1.5 mg/kg was administered. Due to the patient's low platelet count, dual antiplatelet therapy was avoided. Rituximab therapy was permanently discontinued and the patient was monitored closely in the acute cardiac unit. 

Outcome and follow-up 

The patient was hospitalized for five days, during which he was managed for ACS (NSTEMI). His condition stabilized, and no further chest pain occurred. He was discharged to his home, with a follow-up scheduled with the hematology and cardiology teams to ensure appropriate ongoing care and management.

## Conclusions

While it may be a rare occurrence, this case highlights the serious cardiac side effects of using rituximab and therefore should remain within the clinician’s purview. The sudden onset of this chest pain could have cascaded into a more clinically significant issue with potentially detrimental outcomes if the root cause had not been identified and acted upon in a timely manner. Early discontinuation of the offending drug, along with immediate commencement of treatment, significantly improved outcomes. In effect, this management reinforced the causal relationship between rituximab and ACS (NSTEMI); to that extent, close cardiac monitoring during infusion in high-risk patients is highly recommended.

## References

[1] Keswani AN, Williams C, Fuloria J, Polin NM, Jahangir E (2015). Rituximab-induced acute ST elevation myocardial infarction. Ochsner J.

[2] Roy A, Khanna N, Senguttuvan NB (2014). Rituximab-vincristine chemotherapy-induced acute anterior wall myocardial infarction with cardiogenic shock. Tex Heart Inst J.

[3] Arunprasath P, Gobu P, Dubashi B, Satheesh S, Balachander J (2011). Rituximab induced myocardial infarction: a fatal drug reaction. J Cancer Res Ther.

[4] Mostkowska A, Rousseau G, Raynal NJ (2024). Repurposing of rituximab biosimilars to treat B cell mediated autoimmune diseases. FASEB J.

[5] Mahler SA, Hiestand BC, Goff DC Jr, Hoekstra JW, Miller CD (2011). Can the HEART score safely reduce stress testing and cardiac imaging in patients at low risk for major adverse cardiac events?. Crit Pathw Cardiol.

[6] Fernando SM, Tran A, Cheng W (2019). Prognostic accuracy of the HEART score for prediction of major adverse cardiac events in patients presenting with chest pain: a systematic review and meta-analysis. Acad Emerg Med.

[7] Armitage JD, Montero C, Benner A, Armitage JO, Bociek G (2008). Acute coronary syndromes complicating the first infusion of rituximab. Clin Lymphoma Myeloma.

[8] Lee L, Kukreti V (2012). Rituximab-induced coronary vasospasm. Case Rep Hematol.

[9] Cheungpasitporn W, Kopecky SL, Specks U, Bharucha K, Fervenza FC (2017). Non-ischemic cardiomyopathy after rituximab treatment for membranous nephropathy. J Renal Inj Prev.

[10] Patil VB, Lunge SB, Doshi BR (2020). Cardiac side effect of rituximab. Indian J Drugs Dermatol.

[11] Monsuez JJ, Charniot JC, Vignat N, Artigou JY (2010). Cardiac side-effects of cancer chemotherapy. Int J Cardiol.

[12] Cersosimo RJ (2003). Monoclonal antibodies in the treatment of cancer, part 2. Am J Health Syst Pharm.

[13] Verma SK (2016). Updated cardiac concerns with rituximab use: a growing challenge. Indian Heart J.

